# Klinische Ethikberatung in der außerklinischen Langzeitpflege durch den Landesverband Hospiz OÖ – Bericht der ersten 5 Jahre

**DOI:** 10.1007/s10354-020-00801-x

**Published:** 2021-01-13

**Authors:** Kurt Lenz, Helmut Mittendorfer, Helga Sterrer, Arbeitsgruppe klinische Ethikberatung des Landesverbandes Hospiz Oberösterreich, Dietmar Brunschütz, Andrea Glanzer, Daniela Feregyhazy-Astecker, Claudia Kargl, Walter Lamplmayr, Kurt Lenz, Helmut Mittendorfer, Hans Popper, Michael Rosenberger, Helga Sterrer

**Affiliations:** 1 Landesverband Hospiz, Pfalzgasse 2, 4055 Pupping, Österreich; 2 Medizinische Universität Wien, Wien, Österreich

**Keywords:** Geriatrische Langzeitpflege, Ethikberatung, Patientenverfügung, Klinisches Ethikkomitee, Künstliche Ernährung, Nursing home, Ethic consultation, Advanced directives, Clinical ethic comitee, Artificial nutrition

## Abstract

Die Betreuung pflegebedürftiger alter Menschen in Senioreneinrichtungen und Altenheimen stellt deren Mitarbeiter in ihrer täglichen Arbeit immer wieder vor ethischen Fragen. Der Hospizverband Oberösterreich hat vor 5 Jahren ein klinisches Ethikkomitee eingerichtet, deren Aufgabe es ist, Mitarbeiter von außerklinischen Langzeiteinrichtungen bei ethischen Problemstellungen zu beraten und Hilfe zur Lösung gemeinsam zu erarbeiten. In dieser Zeit wurden 38 ethische Beratungen durchgeführt.

Die Anfragen kamen von 24 der 137 in Oberösterreich derzeit bestehenden Langzeiteinrichtungen. Wobei 9 Einrichtungen mehr als eine Anfrage zu einer Beratung in diesem Zeitraum stellten. Teilnehmer an den Beratungen waren einerseits Mitglieder des Behandlungsteams, wobei bei 30 der 38 Beratungen auch die/der behandelnde Ärztin/Arzt anwesend war. Von Seiten des Ethikteams des Hospizverbandes nahmen in der Regel 2 (1–3) Mitglieder teil, wobei ein Mitglied die Moderation leitete, das weitere fertigte das Protokoll an. Die häufigsten Konfliktfelder, die zu einer Beratung führten, waren (bei Mehrfachnennungen) mit über 25 % die Frage nach der Krankenhauseinweisung, gefolgt von Konflikten mit Angehörigen, die Frage der künstlichen Ernährung (einschließlich Setzen bzw. Entfernen einer PEG Sonde) und Einschränkungen der persönlichen Freiheit. Die Hälfte der involvierten Bewohner litten unter einer schweren Demenz, 80 % waren nicht mehr selbstbestimmend. Bei keinem/r der BewohnerInnen lag eine verbindliche Patientenverfügung vor.

## Abstract (en)

Despite the unique nature of ethical issues in a longterm care setting, there is limited help in decision making for nursing home personnel. 2015 the Hospizverband of Upper Austria established a clinical ethic committee to help providing a good quality of decision making in nursing home. A consultation could be asked by the managing nurses of the longterm care institution, by the resident or by relatives of the nursing home residents. Since the start of the ethic committee 24 from 137 nursing homes in Upper Austria asked for a consultation. All consultations came from the personnel of the nursing homes. Beside the nursing team and 1–3 members of the ethic committee, in 75% of the consultations the physician was present. The most often discussed ethical issues were hospital admission (25%), conflicts with relatives of the residents, artificial nutrition (including PEG tube insertion and displacement) and use of restraints. The most underlying illness was dementia (50%). 20% of the residents were competent in their wishes, but none of the involved residents had a written precisely patient decree.

## Einleitung

Ethische Fragestellungen und ethischer Klärungsbedarf im stationären Bereich und im außerklinischen Bereich nehmen sukzessive zu. Es sind Fragen im Zusammenhang mit Grenzen der Behandlung, Differenzen im Verständnis von Fürsorgepflicht und Respekt vor der Patientenautonomie, sowie (Rechts‑) Ansprüchen von Patienten, bzw. zu pflegenden Personen, aber auch Allokationsfragen. In vielen primär angloamerikanischen Ländern, später auch in Deutschland und der Schweiz wurden klinische Ethikkomitees zur Beratung bei diesen Problemen und Fragestellungen gegründet.

In Österreich waren es lokale Initiativen, die ab 2007 zur Etablierung von klinischen Ethikkomitees an der Medizinischen Universität Graz, sowie in den Einrichtungen der Barmherzigen Brüder Österreich und der Vinzenz Gruppe führten. In den letzten Jahren wurden an weiteren Krankenanstalten klinische Ethikkomitees errichtet, ohne dass diese allerdings zurzeit flächendeckend vorhanden sind.

Noch später haben sich Ethikberatungen im Langzeitbereich etabliert. In einer Untersuchung in den USA aus dem Jahre 1988 wurde von 8 % der Langzeitpflegeeinrichtungen, die an der Befragung teilnahmen, über ein lokales Ethikkomitee berichtet [1]. Berechnet auf die Gesamtzahl der Langzeitpflegeeinrichtungen waren dies 2 % aller Einrichtungen. 2006 wurde in Deutschland das Frankfurter Netzwerk Ethik in der Altenpflege begründet. In einer Zusammenstellung aus dem Jahre 2018 bestanden in Deutschland 35 Organisationen, die eine ambulanten Ethikberatung durchführen [2]. In einer Untersuchung aus dem Jahre 2002 in der Schweiz gaben 57 von 393 (18 %) angeschriebenen Institutionen (sowohl Akutkrankenhäuser als auch Pflegeeinrichtungen) an, über eine Ethikkommission zu verfügen [3].

2015 wurde in Rahmen einer Vorstandssitzung des Landesverbandes Hospiz Oberösterreich der Plan einer in Österreich erstmaligen Etablierung einer klinischen Ethikberatung im außerklinischen Bereich, mit einem primären Beginn in der geriatrischen Langzeitpflege, diskutiert. In der Folge bildete sich eine Arbeitsgruppe bestehend aus Experten aus dem Pflegebereich, Palliativbereich, ärztlichen Bereich, Seelsorge und Psychologie. Zusätzlich konnten Vertreter aller in Österreich vertretenen religiösen Gruppen als externe Berater gewonnen werden. Von dieser Arbeitsgruppe wurde in der Folge das Projekt ausgearbeitet und den Heim- und Pflegedienstleitungen der Langzeiteinrichtungen in Oberösterreich im Rahmen deren Regionaltreffen vorgestellt [4]. Als Vorbild dienten einerseits klinische Ethikkommissionen in österreichischen Krankenhäusern, andererseits die bisherigen Erfahrungen derartiger Einrichtungen in Deutschland [5].

Zur Durchführung der Beratungen erfolgte ein zweitägiges Moderatorentraining durch den Medizinethiker Jürgen Wallner [6]. Nach Etablierung einer Ausbildung zur Ethikberatung im Gesundheitswesen in Österreich nach den Richtlinien der Akademie für Ethik in der Medizin (AEM), begannen im Jahre 2020 vier Mitglieder des Hospizethikkomitees diese zusätzliche Ausbildung.

Im Frühjahr 2016 wurden die ersten ethischen Fallberatungen durch Mitglieder des Ethikkomitees des Hospizverbandes durchgeführt. Im Folgenden soll über die ersten Erfahrungen und Ergebnisse dieser ethischen Fallberatungen berichtet werden.

## Methodik

### Anfrage um Durchführung einer ethischen Fallberatung

Jede(r) niedergelassene Ärztin/Arzt, Verantwortliche(r) einer Pflegedienstleitung bzw. Bereichsleitung in Langzeiteinrichtungen, sowie seit 01.01.2020 eines ambulanten Dienstes, kann um die Durchführung einer ethischen Fallberatung anfragen. Die Anfrage sollte per E‑Mail: ethische.fallberatung@gmx.at (ein Anforderungsformular kann vom Landesverband Hospiz OÖ per E.mail vorweg angefordert werden) oder per Telefon an den Landesverband Hospiz Oberösterreich erfolgen. Die Anforderung sollte folgende Punkte enthalten:

- Name der anfordernden Person und der anfordernden Einrichtung, mit
- Telefonnummer und E‑Mail-Adresse sowie Zeiten der telefonischen Erreichbarkeit,
- ethische Fragestellung
- Terminvorschlag (Dringlichkeit ja/nein)

Bewohner bzw. Angehörige von Bewohnern der Einrichtung können über den Erwachsenenschutzverein um eine ethische Beratung durch das Ethikkomitee anfragen.

Von Seiten des Ethikkomitees werden von K.L. und H.M. täglich die eingehenden E‑mail Nachrichten gesichtet, einer der beiden nimmt darauf telefonisch Kontakt mit der anfordernden Person auf und entscheidet, ob ein Konsil initiiert werden soll. Ist die Entscheidung zur Durchführung eines Ethikkonsils gefallen, so wird von K.L. oder H.M. mit der anfordernden Person vereinbart:

- welche Personen an dieser Besprechung teilnehmen
- der Besprechungsort und -zeitpunkt
- die Besprechungsdauer

Von Seiten des Antragsstellers müssen die medizinischen Entscheidungsträger, einschließlich des behandelnden Arztes, sowie die in dem Fall involvierten Mitarbeiter des Hauses eingeladen werden. Patienten bzw. deren Angehörige nehmen nach Absprache an der Sitzung teil. Bei Ansuchen durch Angehörige werden die Entscheidungsträger der Langzeiteinrichtung vom Ethikkomitee verständigt, falls dies nicht schon von dem Erwachsenenschutzverein erfolgt ist.

In den Besprechungen werden die ethischen Fragen des jeweiligen Falles im Lichte medizinischer, pflegerischer, psychologischer, sozialer und seelsorgerischer Umstände und Optionen diskutiert. Es wird ein standardisiertes Vorgehen angelehnt an die Nimwegener Methode [7] unter Verwendung von Formblättern angewendet. Eine derartige Besprechung sollte maximal 2 h dauern. Es besteht die Möglichkeit im Bedarfsfall weitere Besprechungen einzuberufen, falls in dieser Sitzung nicht alle Fragen geklärt werden konnten. Die Entscheidung über das weitere Vorgehen im konkreten Fall liegt ausschließlich innerhalb der jeweiligen Institution.

Das Ergebnis der Beratung wird schriftlich durch den Verantwortlichen des Ethikkomitees zusammengefasst und an den Anfrager übermittelt. Ergebnisberichte sind Bestandteile von Krankenunterlagen bzw. der Bewohnerdokumentation in der Langzeiteinrichtung. Als solche wenden sie sich an alle, die an der Behandlung bzw. Versorgung beteiligt sind und entsprechende Einsichtsrechte in die jeweiligen Unterlagen haben.

Über die Beratung im klinischen Ethikkomitee (Verlauf und Ergebnis) wird absolute Verschwiegenheit gegenüber Dritten bewahrt. Die Beratung erfolgt durch die Mitglieder des Ethikkomitees ehrenamtlich, d. h. der anfordernden Stelle erwachsen keine Kosten.

## Ergebnisse

Im Zeitraum 01.03.2016–01.09.2020 wurden insgesamt 42 Anträge auf Beratungen gestellt (Tab. 1). Die Anträge wurden hauptsächlich von geriatrischen Langzeiteinrichtungen gestellt. Sämtliche Beratungen wurden entweder von der Heimleitung oder der Pflegedienstleitung der Einrichtung angefordert. In 4 Fälle konnte keine Beratung durchgeführt werden, da die Bewohner kurz nach dem Antrag verstorben sind. Die restlichen 38 Anträge wurde in 42 Beratungen diskutiert.

**Table 1:** 

Jahr	Anträge (*n*) und Antragsteller	Konsens in 1. Sitzung	Konsens in 2. Sitzung	Konsens in 3. Sitzung	Vor Beratung verstorben
2016	8 geriatrische Langzeiteinrichtungen	6	1	1	0
2017	7 geriatrische Langzeiteinrichtungen	7	0	0	0
2018	8 geriatrische Langzeiteinrichtungen	6	1	0	1
	2 Einrichtungen für Huntington Erkr.	2	0	0	0
	1 Einrichtung angeborene schwere neurolog. Defizit	1	0	0	0
	1 Einrichtung für geistig abnorme Rechtsbrecher	1	0	0	0
2019	11 geriatrische Langzeiteinrichtungen	8	1	0	2
2020	3 geriatrische Langzeiteinrichtungen	2	0	0	1
	1 Einrichtung für schwere körperliche Beeinträchtigung	1	0	0	0

Das Alter der BewohnerInnen (29 Frauen, 11 Männer), deren Probleme zur Beratung führten, betrug im Durchschnitt 84 (30–93) Jahre. Am häufigsten fand sich als vorherrschende Erkrankung eine Demenz, gefolgt von einem Multiinfarktgeschehen des Gehirns mit massiven neurologischen Ausfällen (Tab. 2).

**Table 2:** 

Erkrankung	Häufigkeit (*n*)
Demenz	19
Multiinfarktgeschehen	10
Multiple Sklerose	3
Chorea Huntington	2
Fortgeschrittene Morbus Parkinson Erkrankung	1
Malignom	1
Amyotrophe Lateralsklerose	1
Infantile Zerebralparese	1

Teilnehmer der Ethikberatungen: bei 34 von 42 der durchgeführten Beratungen war der behandelnde Arzt anwesend, bei 8 Beratungen eine Teilnahme von Seiten des Arztes aus terminlichen Gründen nicht möglich. Von Seiten des Ethikkomitees des Hospizverbandes waren bei 38 Beratungen ein Moderator und ein Schriftführer anwesend, bei 4 Beratungen war der Moderator gleichzeitig Schriftführer.

Die häufigsten Konfliktfelder, die zur Anfrage kamen, waren die Frage der Krankenhauseinweisung (*n* = 12), Konflikte mit Angehörigen (*n* = 11) und künstliche Ernährung (*n* = 11) (Tab. 3).

**Table 3:** 

Konflikt	Häufigkeit (*n*)
Krankenhauseinweisung	12
Konflikt mit Angehörige	11
Künstliche Ernährung (einschließlich PEG Sondenlegung bzw. -entfernung)	11
Zwangsmaßnahmen	10
Aggressionen gegen Pflegende	9
Therapievorenthalt	9
Therapieabbruch	4
Aggression gegen Pflege	4
Selbstbestimmung	3
Konflikt Arzt/Pflege	1
Sterbehilfewunsch der Bewohnerin	1
Andere	1

8 BewohnerInnen (20 %) waren zum Zeitpunkt der Beratung selbstbestimmend, zwei davon hatten die Erstellung einer Patientenverfügung abgelehnt. Eine verbindliche Patientenverfügung war zum Zeitpunkt der Beratung bei keinem der BewohnerInnen vorhanden, bei einem/r bestand eine nicht verbindliche Patientenverfügung. Bei 5 bestand eine Sachwalterschaft (auch für medizinische Fragen), bei 3 eine Vorsorgevollmacht, bei einer Bewohnerin eine gesetzliche Erwachsenenvertretung.

Bei 34 der 38 Beratungen kamen schriftliche Rückmeldungen, die alle die Beratung als sehr hilfreich eingestuft hatten. Bei einer Rückmeldung kam die Anmerkung, dass die schriftliche Dokumentation vor Ort als störend empfunden wurde. Als sehr positives Resultat wurde eine Entlastung aller Beteiligter in dem jeweiligen Konflikt durch die Beratung angeführt, sowie ein „neuer Umgang“ mit dem Problem, vor allem da die Beteiligten das Gefühl hatten, dass sie mit dem Problem nicht alleine gelassen werden.

Fort- und Weiterbildung: Zusätzlich zu diesen Beratungen wurden von den Mitgliedern des Hospizethikkomitees 10 Fortbildungsvorträge zu ethischen Fragestellungen (DNR, neues Erwachsenenschutzgesetz etc.) und 4 Vorträge zum Vorsorgedialog in den einzelnen Langzeiteinrichtungen abgehalten.

## Diskussion

Für viele Menschen entstehen durch die Übersiedelung in eine Langzeiteinrichtung eine Vielzahl von Problemen. Sie müssen die Bedingungen einer Einrichtung und die Handlungsabläufe akzeptieren, die Privatsphäre ist eingeschränkt und auch die Selbstbestimmtheit kann durch fremdbestimmte Anforderungen beeinträchtigt sein. Viele der betroffenen Personen sind nicht (mehr) entscheidungsfähig. Patientenverfügungen sind, wenn überhaupt vorhanden, nicht immer auf die konkrete Situation anwendbar. Eine Ethikberatung soll hierbei helfen, Konflikte zu lösen bzw. die Entstehung zukünftiger Konfliktsituationen möglichst hintanzuhalten. In Oberösterreich werden derzeit in den 7 Regionen in 137 Langzeiteinrichtungen über 12.000 Menschen betreut [8]. Seit Beginn des Ethikkomitees des Hospizverbandes kamen von 24 (18 %) dieser 137 Langzeiteinrichtungen eine Anfrage um Ethikberatung, wobei bei über einem Drittel der ersten Anfrage eine oder mehrere weitere Anfragen folgten. Die Ethikberatungen dauerten in der Regel zwei Stunden. Die Thematik der Konfliktsituationen war sehr ähnlich zu den Erfahrungen aus anderen Ländern [1, 5, 9]. So fanden sich in unserer Untersuchung bei keiner/m der involvierten BewohnerInnen eine verbindliche Patientenverfügung, von zwei BewohnerInnen wurde eine Ausstellung einer Patientenverfügung sogar abgelehnt, ohne dass letztendlich die Gründe hierfür geklärt werden konnten. Bei einer Bewohnerin bestand eine nicht verbindliche (nach früherer Definition beachtliche Patientenverfügung), die jedoch sehr allgemein gehalten war und dadurch zur Lösung des Problems nicht von relevanter Hilfe war. Auch die Wünsche und Erwartungen der Angehörigen sind vielschichtig und reichen von maximalen Therapiewünschen bis hin zu dem Wunsch nach sofortiger Therapieeinstellung. Nicht selten sind Angehörige von Informationen über die Erkrankung aus verschiedensten Quellen überflutet, wodurch sich zusätzliche Kommunikationsprobleme und unterschiedliche Auffassungen sowohl innerhalb der Familie der Angehörigen als auch zwischen dem Behandlungsteam und den Angehörigen ergeben können. In unserer Untersuchung waren es vor allem Bewohner mit Demenz, mit den daraus sich ergebenden Problemen der Aggression gegen die Pflegenden, der Frage nach Zwangsmaßnahmen, der Frage der Selbstbestimmung – die besonders bei dementen BewohnerInnen mit ausgeprägtem Bewegungsdrang mit dem Problem der Selbstgefährdung einherging. Weiter fanden sich auch sehr häufig Konflikte der Pflegenden mit Angehörigen, bei denen oft Schuldgefühle gegenüber ihren dementen Liebsten zu einer Überfürsorge führten, die eine sorgfältige Pflege und vor allem auch gerechte Pflege gegenüber den anderen Heimbewohnern massiv erschwerte [10]. Sehr wirksam hat sich erwiesen, Hilfe bei der Erstellung von Grenzen anzubieten, wodurch einerseits den Pflegenden Sicherheit in Umgang mit den Demenzkranken gegeben wurde, andererseits sich auch für Angehörige eine Nachvollziehbarkeit der Handlungen ergaben. Auffallend war eine sehr niedrige Zahl an vorausschauenden Entscheidungen wie verbindliche Patientenverfügung oder Vorsorgevollmacht. Ähnliche Zahlen konnten in einer Untersuchung an Intensivpatienten in Österreich erhoben werden [11]. Möglicherweise kann durch das verbesserte nun vorliegende Erwachsenenschutzgesetz [12] mit den Möglichkeiten der Installation von Erwachsenenvertretern bei eingeschränkt kompetenten Personen eine Verbesserung dieser Problematik erzielt werden. Zusätzlich wurde in den letzten Jahren die Implementierung des Vorsorgedialogs in Langzeiteinrichtungen begonnen [13]. Die dadurch ermöglichte vorrausschauende Planung durch Erfassung der Wünsche der BewohnerInnen bereits bei Heimaufnahme sollte in Zukunft das Auftreten von ethischen Problemen weiter vermindern.

Zu den Voraussetzungen einer Langzeiteinrichtung gehört das besondere Bemühen der Pflegenden um das Wohl des zu Pflegenden und dem Respekt vor der Autonomie der Bewohner. Zwischen den Bewohnern und den Pflegenden entwickeln sich daher hochkomplexe Beziehungen. Aus diesen unterschiedlichen Faktoren entstehen für die behandelnden Berufsgruppen Belastungen und Konfliktsituationen. Hilfe gibt in solchen Fällen eine ethische Fallberatung als ein wichtiges Instrument zur Implementierung einer ethischen Reflexionskultur. Ein Großteil der von uns beobachteten Fälle waren einerseits Aggressionen gegen die Pflegenden, andererseits eine massive Fluchttendenz der BewohnerInnen, die zu Behandler/Pflegenden Konfliktsituationen führte. Die Moderatoren einer Ethikberatung, die besonders geschult sind, haben hierbei selbst keine Entscheidung zu treffen, jedoch die Aufgabe Lösungsmöglichkeiten anzuregen bzw. an der Erstellung mitzuhelfen und gegebenenfalls Hinweise zum rechtlichen Rahmen zu geben. Eine Ethikberatung schreibt damit den beteiligten Personen ihr Handeln nicht vor; die rechtliche Verantwortung verbleibt bei den handelnden Personen, die Ethikberatung stellt eine Unterstützung in der Entscheidungsfindung dar.

Eine Ethikberatung wurde in einer Untersuchung aus Deutschland von allen Beteiligten grundsätzlich als hilfreich erlebt und zunehmend als wichtiger Qualitätsindikator der (ambulanten) Versorgungsstruktur angesehen [14]. Es wurde hierbei über eine Entlastung für alle Beteiligten in einer konfliktbehafteten Situation, eine Sensibilisierung der Mitarbeiter gegenüber ethischen Fragestellungen mit positiven Veränderungen der Kommunikationsstrukturen berichtet. Auch die angemessene Aufmerksamkeit der Beteiligten wurde hier hervorgehoben.

Ähnlich diesen Berichten aus anderen Ländern ergaben auch bei unseren Nachbefragungen sehr positive Effekte, vor allem sei es zu einer Sensibilisierung gegenüber den ethischen Fragenstellungen von Seiten der Pflegenden gekommen, einhergehend mit positiven Veränderungen der Kommunikation mit den zu Pflegenden, aber auch unter den Mitarbeitern. Aber auch das Gefühl, dass ihr Problem ernst genommen worden ist und sie nicht damit alleine gelassen werden, führte zu einer Erleichterung und Entlastung der betroffenen Mitarbeiter. Ebenso fühlte sich zumindest ein Teil der Angehörigen mit ihren Problemen ernst genommen, wodurch sich der Konflikt Angehörige/Pflegende entspannte und die tägliche Arbeit der Pflegenden leichter wurde.

## Konklusion

2015 erfolgte die Konstituierung eines klinischen Ethikkomitees für den außerklinischen Bereich organisiert über den Hospizverband OÖ. Klinische Ethikkomitees sind multidisziplinär besetzte Kleingremien zur Unterstützung bei ethisch relevanten Fragen, die einer vertieften multiperspektivischen Erörterung bedürfen. Sie fungieren nicht als Entscheidungs-, sondern als Beratungsorgan mit dem Ziel der Erstellung von Empfehlungen für eine ethisch gerechtfertigte Vorgehensweise in strittigen oder problematischen Fällen. Dem klinischen Ethikkomitee des Hospizverbandes OÖ gehören Ärzte verschiedener Fachrichtungen, einschließlich Palliativmedizin, Pflegefachkräfte, Psychologen, Juristen und Vertreter von Religionsgemeinschaften, an. Die Mitglieder dieser Gruppe führen diese Beratungen ehrenamtlich durch, deren fachliche Kompetenz ist durch eine spezielle Moderatorenausbildung für Ethikberatungen, bzw. zusätzliche Absolvierung des Masterstudiums in Medizin- und Bioethik gegeben. Bislang wurde eine Beratung durch dieses Ethikkomitee von 24 Langzeiteinrichtungen in Oberösterreich in Anspruch genommen. Durch diese ethische Beratungstätigkeit konnten einerseits Lösungen erarbeitet werden, wodurch die tägliche Arbeit der Pflegenden erleichtert werden konnte, andererseits kam es zu einer Sensibilisierung gegenüber ethischen Fragenstellungen, wodurch eine Entstehung zukünftiger ethischer Probleme frühzeitig erkannt werden kann. Unterstützt wird dies durch Vorträge zu verschiedenen ethischen Problemen in den Einrichtungen. In Zukunft werden zusätzlich zum stationären Bereich diese Beratungen auch dem ambulanten außerklinischen Bereich, wie ambulantes Palliativteam oder Hauskrankenpflege, angeboten werden.
